# Linoleate-Rich Safflower Oil Diet Increases Linoleate-Derived Bioactive Lipid Mediators in Plasma, and Brown and White Adipose Depots of Healthy Mice

**DOI:** 10.3390/metabo12080743

**Published:** 2022-08-12

**Authors:** Deena B. Snoke, Austin Angelotti, Kamil Borkowski, Rachel M. Cole, John W. Newman, Martha A. Belury

**Affiliations:** 1 Deparment of Medicine, Larner College of Medicine, Univeristy of Vermont, Burlington, VT 05405, USA; 2 Department of Human Sciences, College of Education and Human Ecology, The Ohio State University, Columbus, OH 43212, USA; 3 Interdisciplinary PhD Program in Nutrition, The Ohio State University, Columbus, OH 43210, USA; 4 West Coast Metabolomics Center, Genome Center, University of California Davis, Davis, CA 915616, USA; 5 Western Human Nutrition Research Center, United States Department of Agriculture, Agriculture Research Service, Davis, CA 95616, USA; 6 Department of Nutrition, University of California Davis, Davis, CA 915616, USA

**Keywords:** polyunsaturated fatty acids, oxylipins, endocannabinoids, lipid mediators, dietary fats, adipose tissue, metabolic disease

## Abstract

Polyunsaturated fats are energy substrates and precursors to the biosynthesis of lipid mediators of cellular processes. Adipose tissue not only provides energy storage, but influences whole-body energy metabolism through endocrine functions. How diet influences adipose–lipid mediator balance may have broad impacts on energy metabolism. To determine how dietary lipid sources modulate brown and white adipose tissue and plasma lipid mediators, mice were fed low-fat (15% kcal fat) isocaloric diets, containing either palm oil (POLF) or linoleate-rich safflower oil (SOLF). Baseline and post body weight, adiposity, and 2-week and post fasting blood glucose were measured and lipid mediators were profiled in plasma, and inguinal white and interscapular brown adipose tissues. We identified over 30 species of altered lipid mediators between diets and found that these changes were unique to each tissue. We identified changes to lipid mediators with known functional roles in the regulation of adipose tissue expansion and function, and found that there was a relationship between the average fold difference in lipid mediators between brown adipose tissue and plasma in mice consuming the SOLF diet. Our findings emphasize that even with a low-fat diet, dietary fat quality has a profound effect on lipid mediator profiles in adipose tissues and plasma.

## 1. Introduction

Dietary fat quality has been demonstrated to have an enormous impact on the onset and progression of metabolic health conditions associated with dysregulated adipose tissue function and dyslipidemia [1,2,3]. At the cellular level, dietary fats are not only energy-rich fuel substrates, but also serve a structural role in membrane phospholipids and are signaling molecules regulating energy metabolism and other cellular processes [4,5,6]. Dietary polyunsaturated fatty acids (PUFAs) are precursors for both oxylipins (e.g., bioactive oxygenated lipids) and the endocannabinoid and endocannabinoid-like acylethanolamides and monoacylglycerols [7,8]. The most well-characterized of these lipid mediators include those derived from arachidonic acid (AA; 20:4*n*6), and its precursors dihomo-γ-linoleic acid (DGLA; 20:3*n*6) and the essential fatty acid linoleic acid (LA; 18:2*n*6). Various lipid mediators derived from LA, AA, and DGLA have been implicated as both protective and detrimental to disease states, including, but not limited to, cardiovascular disease, aging, Alzheimer’s disease, obesity, and type 2 diabetes mellitus [9,10], supporting the importance of a physiological balance between pro- and anti-inflammatory mediators in the manifestation of cardiometabolic diseases.

Over the past two decades, our understanding of the function of adipose tissue has expanded beyond its utility as an energy storage depot. Adipocyte receptors monitor blood levels of glucose, hormones, and inflammatory cytokines, and adipose-tissue-resident cells are responsible for the production and secretion of adipokines, lipids, and other inflammatory signals through a variety of physiological functions [11]. Thus, adipose tissues have the profound ability to manage whole-body energy metabolism through their endocrine functions, and have been implicated in a number of conditions of metabolic dysregulation, including those mentioned above [11]. Of note, recent studies have explored the notion that LA-derived bioactive lipid mediators from brown adipose tissue (BAT) act as adipokines to improve insulin sensitivity and metabolic function in skeletal [12] and cardiac muscles [13], suggesting that the endocrine functions of adipose tissue may also include other bioactive lipid mediators.

In adipose tissue, PUFA-derived lipid mediators influence adipogenesis, mainly through peroxisome-proliferator-activated receptor (PPAR) and G-protein-coupled receptor-mediated processes [14]. However, when compared to diets rich in saturated fats (SFA), high PUFA diets, rich in either omega-3 or omega-6 lipids, have protective effects on the progression of metabolic diseases that may include the modulation of adipose function [15,16,17]. However, less is known about how dietary SFA and PUFA interact to influence adipose tissue lipid mediator levels, and how this corresponds to the presence of lipid mediators in circulation.

In an exploratory notion, we aimed to compare the local and systemic effect of diets containing palm oil and LA-rich safflower oil on species of lipid mediators in inguinal white adipose tissue (iWAT), BAT, and plasma from healthy mice. Palm oil is rich in SFA and monounsaturated oleic acid, and contains low quantities of PUFAs. Due to its low production cost [18] and desired lipid profile for shelf-stable foods, the use of this oil continues to rise to meet global demand [19], and is becoming highly incorporated into the human food supply chain [18]. A recent meta-analysis reported contradicting findings regarding the health effects of increased palm oil consumption, and announced a need for well-designed studies addressing this question [20]. In response, we have compared a palm-oil-rich diet with an LA-rich diet, the most commonly consumed dietary PUFAs with demonstrated positive metabolic effects [21,22]. Thus, these diets were intended to explore the extent by which *n*-6 PUFA-derived lipid mediators could be modulated in BAT, WAT, and plasma of healthy animals when consuming a quantity of dietary fat that is typical of rodent diets.

To address this research question, wild-type mice were fed diets low in fat (6% fat by weight; 15% kcal), differing only in their source and lipid composition (palm oil, LA-rich safflower oil), for a period of 5 weeks, and their adipose tissue and plasma oxylipins and endocannabinoids were measured. Here, we report the composition of tissue-derived lipid mediators in each respective tissue. Then, we assess the relationship between adipose and plasma oxylipins, and physiological measurements collected during the study, and compare similarities in the dietary modulation of lipid mediators between the three tissues analyzed.

## 2. Materials and Methods

### 2.1. Dietary Oil Fatty Acid Analysis

Fatty acid composition of palm oil and LA-rich safflower oil used in POLF and SOLF diets, respectively, were measured using gas chromatography after total lipid extraction with 2:1 (*v*/*v*) chloroform: methanol using Folch et al. methodology [23]. Fatty acid methyl esters were prepared with 5% hydrochloric acid in methanol [24], and analyzed via gas chromatography (Shimadzu, Columbia, MD, USA) using a 30-m OmegawaxTM 320 fused silica capillary column (Supelco, Bellefonte, PA, USA) under previously established conditions [22].

### 2.2. Experimental Animals, Diets, and Study Design

**Figure 1:**
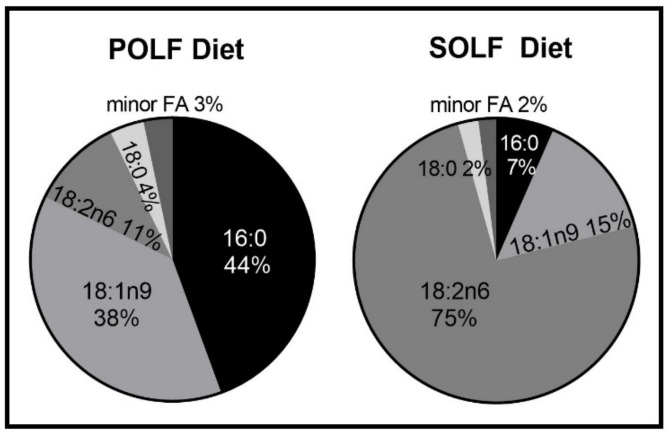
Dietary fatty acid composition of the oils used in the POLF and SOLF diets. Dietary oils (*n* = 3 samples/group) were analyzed by gas chromatography, expressed as percent of total fatty acids. All samples have a coefficient of variation of <3% between experimental replicates.

After consuming a washout diet for 1 week, 10–12-week-old male C57BL6/J mice (*n* = 7–9/group) were weighed and assigned to one of two AIN-93M diets, containing either palm oil or LA-rich safflower oil as the main fat source (Figure 1). The 10–12-week-old male C57Bl6/J mice were obtained from Jackson Laboratory (Bar Harbor, ME, USA) and acclimated to their new environment for 7 days while on a modified washout AIN-93M diet containing equal amounts of the oils used in the study. To monitor food intake and body weight, mice were housed individually with enrichment at 22 +/− 5 °C on a 12:12 h light–dark cycle. Body weight and food intake were measured biweekly. Seven days after the beginning baseline diets, mice began one of two modified AIN-93M isocaloric diets containing 6 wt% (15% kcal) palm oil (POLF diet) or LA-rich safflower oil (SOLF diet; Research Diets, Inc, New Brunswick, NJ, USA). The compositions of the two experimental diets are found in Table S3. To confirm the primary fatty acids present in the dietary oils, the fatty acid compositions of these oils were determined by gas chromatography; these are reported in Figure 1.

On Day 36, mice were sacrificed by cardiac puncture under isoflurane anesthesia with cervical dislocation. Gastrocnemius, adipose depots were collected, weighed, and flash frozen in liquid nitrogen to prepare for analyses (described below), and stored at −80 °C. All procedures were in accordance with institutional guidelines and approved by the Institutional Animal Care and Use Committee at The Ohio State University.

### 2.3. Measurement of Fasting Blood Glucose

Blood glucose was measured after a 5-h fast on Day 35 by pricking the tip of the tail with a 20 g needle, and was quantified using the OneTouch^®^ Ultra^®^ glucose meter (LifeScan Inc., Milpitas, CA, USA).

### 2.4. Plasma Adiponectin and High-Molecular-Weight (HMW) Adiponectin

The concentration of plasma IL-6 (Invitrogen, Carlsbad, CA, USA), and HMW- and total adiponectin (Alpco, Salem, NH, USA) were measured by ELISA according to the manufacturer’s protocols.

### 2.5. EchoMRI for Body Composition

To assess changes in body composition of live mice during the diet treatment paradigm, EchoMRI (Houston, TX, USA) was used at two time points: Day 0 (beginning of diet treatment) and Day 36 (Week 5; day of necropsy).

### 2.6. Targeted Lipidomic and Quantification of Adipose Tissue and Plasma Lipid Mediators

Plasma concentrations of nonesterified PUFA, oxylipins, and endocannabinoids were quantified in 50 µL of plasma or ~30 mg of adipose tissue by liquid chromatography–tandem mass spectrometry (LC-MS/MS) after protein precipitation in the presence of deuterated metabolite analogs (i.e., analytical surrogates) [25]. All samples were processed with rigorous quality control measures, including case/control randomization, and the analysis of batch blanks and pooled matrix replicates. The majority of analytes were quantified against analytical standards, with the exception of eicosapentaenoyl ethanolamide (EPEA), palmitoleoyl ethanolamide (POEA), and the measured PUFAs (i.e., linoleic acid (LA); alpha-linolenic acid (aLA); arachidonic acid (AA); eicosapentaenoic acid (EPA); docosahexaenoic acid (DHA)). For these compounds, area counts were recorded, adjusted for deuterated-surrogate responses, and the relative response factors were expressed as the relative abundance across all analyzed samples.

### 2.7. Statistical Analysis

Body weight, adiposity, and glucose data are represented as the mean ± standard error of the mean (SEM). Plasma adiponectin and glucose data were log-transformed for normalization. Lipid mediator data are presented as the mean ± 95% confidence interval, and average fold differences were calculated (Table 1). For each outcome at a single time point, changes between POLF and SOLF diet groups were analyzed by Student’s *t*-test. For outcomes compared at multiple time points, paired *t*-tests were used to distinguish changes over time for diet consumption. These tests were performed using STATA (StataCorp LLC, College Station, TX, USA) and visualized/graphed with GraphPad Prism (GraphPad Software, San Diego, CA, USA); *p* < 0.05 was considered statistically significant.

Partial least squares discriminant analysis (PLS-DA) was used to identify diet-related differences in metabolite levels in BAT, WAT, and plasma. The PLS-DA model was built using the nonlinear iterative partial least squares algorithm with leave-one-out cross-validation (JMP, SAS institute, Carry, NC, USA), and included all variables for plasma, BAT, and WAT. For clarity purposes, only variables with a variable importance in projection (VIP) score >1.2 were displayed on the loading plot.

Spearman’s rank order correlation was used to determine associations between tissue and plasma metabolites, and clinical measures, including body mass, adiposity, and adiponectin.

## 3. Results

### 3.1. Effect of Palm-Oil-Rich and Linoleate-Rich Safflower Oil Low-Fat Diets on Physiological Parameters

**Figure 2:**
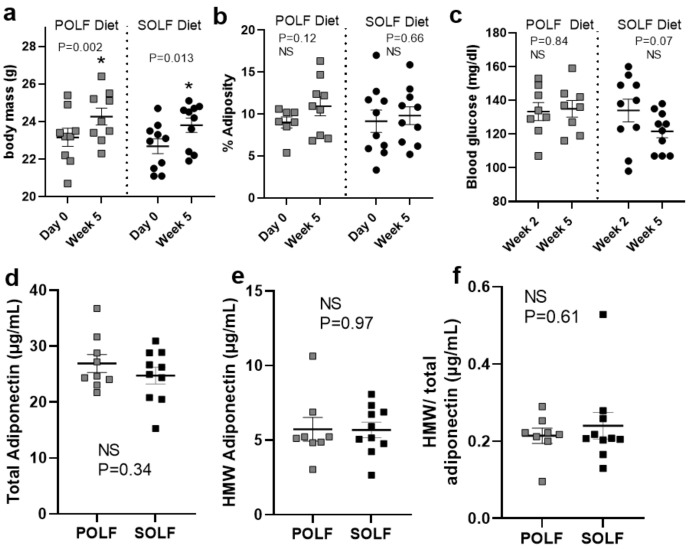
Physiological parameters of mice consuming POLF and SOLF diets for a 5-week period. (**a**) Change in body mass from Day 0 to Week 5, grouped by diet. (**b**) Percent adiposity as measured by EchoMRI from Day 0 to Week 5, grouped by diet. (**c**) Blood glucose measurements on Week 2 and Week 5, grouped by diet. (**a**–**c**) For each respective diet, a paired *t*-test was used to determine significant changes between measurements at the beginning and end of the study. (**d**) Total adiponectin and (**e**) high-molecular-weight adiponectin measured in plasma of mice after 5 weeks on the diet, as well as (**c**) the ratio of HMW/total adiponectin. (**a**–**c**) A paired *t*-test was used to determine changes between Day 0 and Week 5 within each diet group, while ANCOVA analysis was used to compare changes over time between groups and determine any significant differences between slopes over time. (**d**–**f**) Student’s *t*-tests were used to determine significant differences between diet groups at a single timepoint. An asterisk indicates *p* < 0.05 for (**a**–**c**) paired *t*-tests and (**d**–**f**) student’s *t*-tests, respectively. ‘NS’ indicates that *p* ≥ 0.05 and is non-significant for all analyses. While no significant differences in ANCOVA analysis and Student’s *t*-tests were determined. N = 8–10/group.

Short-term consumption of the palm-oil-rich low-fat diet (POLF) and high-LA safflower oil-rich diet (SOLF) diet did not differentially effect body weight, adiposity, or blood glucose levels. After a 5-week consumption period of POLF (SFA:MUFA:PUFA ratio of 4:3:1) or SOLF (SFA:MUFA:PUFA ratio of 1:1:5; see Figure 1 and Table S1 for diet composition), mice increased body weight to a similar extent (Figure 2a), and showed stable and comparable adiposity (Figure 2b) and blood glucose levels (Figure 2c). Additionally, plasma levels of total adiponectin, an adipokine with well-established roles in regulating insulin sensitivity, inflammation, and circulating glucose and lipids [26,27,28,29], were measured. We found that total adiponectin and its most bioactive multimeric form, high-molecular-weight (HMW) adiponectin [26,30], were unchanged between diet groups at the end of the study (Figure 2d–f).

### 3.2. Impact of POLF and SOLF Diets on Brown and White Adipose Tissue and Plasma Lipid Mediators

#### 3.2.1. Univariate Analysis

Experimental diets differentially impacted oxylipin and endocannabinoid levels in BAT, iWAT, and plasma, suggesting global enrichment in LA- and AA-derived lipid mediators in mice fed the SOLF diet. We compared fasting plasma oxylipin profiles with BAT and iWAT tissues after 5 weeks of POLF or SOLF diet consumption. In BAT and iWAT, out of 53 lipid mediators measured, 38% and 47%, respectively, were higher in mice consuming the SOLF diet compared to the POLF diet, the majority of which were LA- or AA-derived. However, no lipid mediators were decreased. In plasma, 36% of lipid mediators were higher in the SOLF diet, which were all LA- or AA-derived, while only two of the lipid mediators measured (3.6%) were lower compared to the POLF diet: 13-hydroxyoctadecatrienoic acid (13-HOTE) and oleoylethanolamide (OEA; Table 1). Overall, oxylipins derived from LA and AA were higher in BAT, iWAT, and plasma of mice fed the SOLF diet compared to those fed the POLF diet. This was not limited to a specific enzymatic pathway; rather, LA- and AA-derived oxylipins were increased in a pathway-independent manner. Conversely, omega-3 PUFA metabolites were largely unchanged (Supplementary Table S2).

Of those lipid mediators measured, some metabolites exhibited tissue-specific changes. Of note, 6-keto-prostaglandin F1a was higher in BAT and WAT, but unchanged in plasma. Out of those mediators measured, only plasma levels of OEA were lower in mice consuming the SOLF diet. Although nonsignificant, plasma 9-hydroxyeicosatetraenoic acid (HETE) was unchanged, while in adipose tissues of mice consuming the POLF diet, it was twofold higher. Similarly, 8,15-DiHETE was not observed in plasma, but is about twofold higher in adipose tissues. Finally, we observed that 9,12,13-trihydroxyoctadecenoic acid (TriHOME) was threefold higher in BAT, but unchanged in iWAT.

#### 3.2.2. Multivariate Analysis

**Figure 3:**
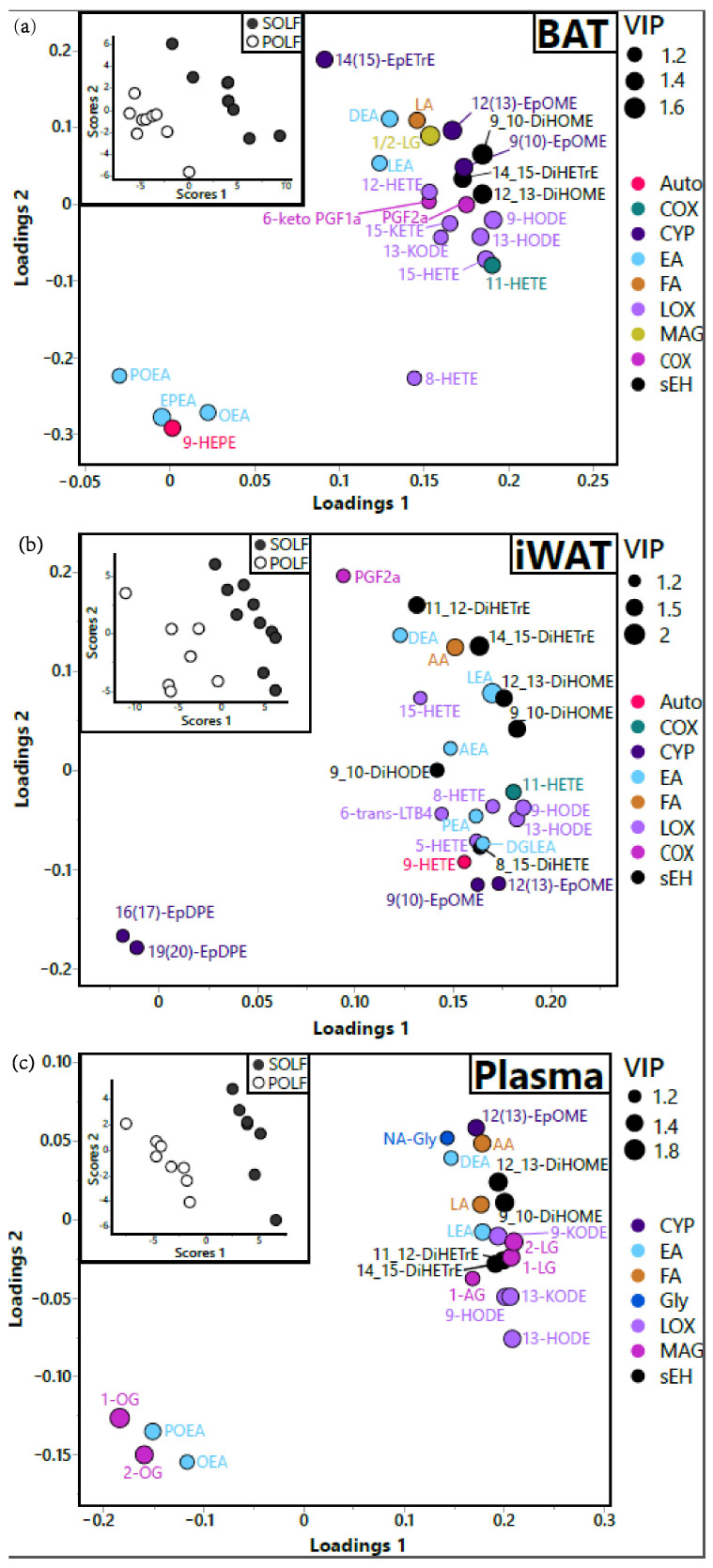
Influence of dietary fat on intratissue lipid mediator speciation in: (**a**) BAT, (**b**) iWAT, and (**c**) plasma. Partial least squares discriminant analysis (PLS-DA) of SOLF vs. POLF. Treatment group discrimination is shown by the SCORES panel (inset), while metabolite weightings in group discrimination are shown by the LOADINGS panel. Loading node color indicates metabolite enzymatic origin. Loading node size indicates metabolite variable importance in projection (i.e., VIP). Analysis was performed with all measured metabolites, but only those with VIP ≥ 1.2 are displayed for clarity purposes. N = 6–9 per group. BAT X^2^ = 0.98, Y^2^ = 1.22, Q^2^ = 0.73; iWAT X^2^ = 0.17, Y^2^ = 0.15, Q^2^ = 0.65; plasma X^2^ = 1.3, Y^2^ = 0.095, Q^2^ = 0.76.

Consistent with the univariate analyses, partial least squares discriminant analysis (PLS-DA) of lipid mediator profiles clearly segregated the two diet groups (Figure 3a–c; Q^2^ > 0.4), with BAT discrimination (Q^2^ = 0.87) being better than iWAT (Q^2^ = 0.84). In BAT, the animals on the SOLF diet showed higher levels of a variety of n6-PUFA-derived lipids, including linoleate itself, linoleoylethanolamide (LEA), 1- and 2-linoleoyglycerol (1/2-LG), hydroxyocdadecaenoic acids (HODEs), keto-octadecanoeic acids (KODEs), epoxyoctadecamonoenoic acids (EpOMEs), and dihydroxyoctadecanoic acids (DiHOMEs), as well as prostaglandin F2alpha (PGF2a), 6-keto-PGF1a, and various HETEs. In contrast, the POLF-diet animals had higher levels of multiple N-acylethanolamines, including oleoylethanolamide (OEA), palmitoleoylethanolamide (POEA), and eicosapentaenoylethanolamide (EPAEA). Additionally, LA-derived metabolites were the main pool of lipid mediators higher in the SOLF diet compared to the POLF diet (Figure 3a), highlighted by enrichment in LA-derived LOX, PLD (EA), and soluble epoxide hydrolase (sEH)-derived metabolites (Figure 3a). Notably, AA-derived prostaglandins PGF2a and 6-keto-PGF1a were also higher in the SOLF diet (Figure 3a).

Similar to BAT, higher DiHOMEs and LOX-derived HODE and KODE metabolites were observed in iWAT (Figure 3b). In contrast, iWAT exhibited higher quantities of AA and its metabolites, and lower DHA epoxides in mice consuming the SOLF diet (Figure 3b). Similar to BAT, mice consuming the SOLF diet exhibited increased LEA and DEA, but this pool of ethanolamides that were higher also included AA-derived arachidonylethanolamide (AEA) and DGLA-derived dihomogammalinoleoylethanolamide (DGLEA; Figure 3b). Notably, despite lower dietary levels of palmitate (16:0), iWAT palmitoylethanolamide (PEA) was higher in the SOLF group (Figure 3b). Only two lipid mediators were higher in the POLF group compared to the SOLF group: 16(17)- and 19(20)-expoxydocosapentanoic acids (EpODE; Figure 3b).

In plasma, the SOLF group had higher LA and AA, along with many of their metabolites, including the LA- and AA-derived monoacylglyerols 1/2-LG and 1-AG, HODEs, EpOMEs, DiHOMEs, dihydroxyeicosatetraenoic acids (DiHETrEs), and LEA. In contrast, the POLF group showed higher oleic-acid-derived monoacylglyerols 1/2-OG, and OEA and POEA (Figure 3c). Of lipid mediator metabolites themselves, the remaining differences between diets were attributed primarily to LA-derived sEH and LOX metabolites (Figure 3c).

### 3.3. Diet-Independent Changes in Lipid Mediators and Relationship to Physiological Measures

Due to the known relationship between LA-derived lipid mediators (e.g., 12,13-DiHOME) and metabolic function in preclinical animal models, we explored diet-independent and cross-tissue relationships between BAT, plasma, and iWAT lipid mediators with lean mass, adiposity, blood glucose, and adiponectin in healthy mice consuming POLF and SOLF diets by Spearman’s rank order correlation (Table S3). In BAT, strong negative correlations were observed between percent adiposity and 12/15-LOX metabolites, including AA-derived 12-HETE (ρ = −0.63), EPA-derived 12-HEPE (ρ = −0.62), and DHA-derived 14- and 17-HDoHE (ρ = −0.77 and −0.74 respectively), in addition to aLA-derived 15,16-DiHODE (ρ = −0.56) and 13-HOTE (ρ = −0.57), and PEA (ρ = −0.65) and AA-derived monoacylglycerol (1/2-AG) (ρ = −0.56).

In iWAT, metabolites from cyclooxygenase (COX) and soluble epoxide hydrolase (sEH) pathways were negatively correlated with percent adiposity. These included: AA-derived prostaglandins PGE2 (ρ = −0.61) and PGF1a (ρ = −0.59), LA-derived 12,13-DiHOME (ρ = −0.57), and AA-derived 8,9, 11,12 and 14,15-DiHETrE (ρ = −0.67, −60, and −65, respectively). Additionally, ½-LG was negatively correlated with percent adiposity.

In plasma, only 1/2-OG was negatively correlated with percent adiposity (ρ = −0.60). Additionally, a negative association between plasma adiponectin and plasma AA and DHA-derived 5-LOX metabolites—5-HETE and 4-HDoHE—(ρ = −63 and −87, respectively) was observed.

### 3.4. Diet-Dependent Differences in BAT and Plasma Lipid Mediator Levels Are Strongly Correlated

**Figure 4:**
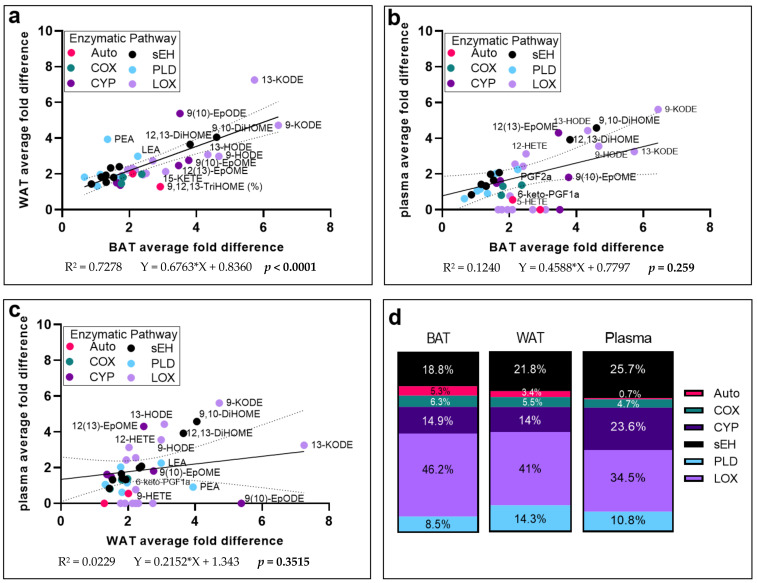
Correlations of average fold difference of mice consuming the SOLF diet compared to the POLF diet among brown adipose tissue, white adipose tissue, and plasma. Average fold difference was calculated for those lipid mediators that were changed by diet in univariate analysis in at least one of the three tissues reported. Average fold difference (reported in Table 1) was calculated by dividing the average of each metabolite in the SOLF diet by that of the POLF diet. Regression analysis was used to determine similarities in fold changes in (**a**) WAT vs. BAT, (**b**) BAT vs. plasma, and (**c**) WAT vs. plasma, reported here with 95% confidence intervals. Those metabolites not detected in one of the two tissues was included as a value of 0. *p* < 0.05 indicates a correlation significantly different from zero. (**d**) The sum of the average fold differences for each tissue was computed and the contributions of each metabolic pathway to the total fold change was determined. Enzymatic pathway abbreviations: COX—cyclooxygenase; LOX—lipoxygenase; Auto—auto-oxidation; CYP—cytochrome P450; sEH—soluble epoxide hydrolase; ADH—alcohol dehydrogenase; PLD—phospholipase D.

To better understand the influence of dietary fat on tissue-specific and global alterations in oxylipin and endocannabinoid-like metabolites, we calculated the average fold difference for those oxylipins exhibiting differences between diets in at least one of the three tissues measured, and looked at the linear relationships between these differences in each tissue, respectively (Figure 4). Positive correlations were observed in the average diet-dependent fold differences of metabolites between BAT and iWAT (*p* < 0.001), and between BAT and plasma (*p* = 0.03; Figure 4a,b). However, the relationship between plasma and iWAT metabolites did not reach the *p* <0.05 level (*p* = 0.35; Figure 4c).

While modest differences were reported compared to other metabolites, the SOLF diet elicited the most similar fold differences in metabolites within soluble epoxide hydrolase (sEH) for all three tissues (Figure 4a–c). Additionally, LA-derived long-chain fatty acid alcohol dehydrogenase-dependent downstream metabolites of HODES, 9-KODE and 13-KODE, exhibited some of the largest fold differences in all three tissues (Figure 4a–c). Of note, in all three tissues, LOX-derived metabolites appear to be most variable between tissues compared (Figure 4a–c).

To visualize the enzymatic pathways contributing to the greatest average fold differences between tissues, the sum of the total fold differences was calculated, and the contributions of each metabolic pathway to total fold differences were visualized as a percent of the total fold differences (Figure 4d; Table S3). To further explore these relationships statistically, the average fold difference for each enzymatic pathway was calculated and compared by one-way ANOVA with Tukey’s post hoc test to look at the effect of tissue on fold differences (Table S4). Only those pathways contributing to small percentages of the total fold differences, i.e., auto-oxidated products (<6%) and COX pathway products (4–7%; Figure 4d), differed between BAT and plasma, with BAT exhibiting the highest relative amounts of COX-mediated and auto-oxidated product differences, and plasma having the lowest (*p* = 0.04 and 0.04, respectively; Table S4).

## 4. Discussion

In this study, we aimed to identify tissue-specific and systemic effects of low-fat diets containing palm oil (POLF diet) and LA-rich safflower oil (SOLF diet) on oxylipin and endocannabinoid-like lipid mediator profiles in BAT, iWAT, and plasma. To address this aim, healthy wild-type mice were fed POLF and SOLF diets for a period of 5 weeks, and physiological measurements were collected in addition to measurements of tissue lipid mediators. Our finding that no distinguishable differences in physiological measurements were observed, aside from a near-significant effect of SOLF diet leading to decreased glucose levels, over the course of the study suggest that we have captured an early stage of diet intervention which, if continued over time, might result in measurable changes in metabolic health.

Even prior to diet-intervention-induced physiological changes, underlying alterations to metabolic processes may occur at the tissue and cellular level. The current study allowed us to identify changes to oxylipins and endocannabinoid-like mediators after POLF and SOLF diet consumption, independent of alterations to body composition and weight, which alone can influence changes to metabolic status. To our knowledge, aside from one study exploring the differences in oxylipin metabolites among white adipose tissues of rats during LA and ALA supplementation [31], this is the first study to provide an in-depth comparison of lipid mediator responses to either high palmitate/oleate–low LA or low palmitate/oleate–high-LA diets among brown and white adipose tissues. We observed global enrichment in LA- and AA-derived lipid mediators: more than 1/3 of the total oxylipins measured in each tissue were higher in mice consuming the SOLF diet, underscoring the fact that even with a low-fat diet, dietary fat quality can have profound effects on lipid mediator profiles in adipose tissues and plasma.

Through univariate and multivariate analyses, we identified tissue-specific differences in lipid mediators influenced by diet. Because BAT produces heat through mitochondrial uncoupling, it is rich in mitochondria. LA is selectively incorporated into cardiolipin, a phospholipid of the mitochondrial membrane [5]. Therefore, it is unsurprising that BAT exhibited higher LA-derived metabolites in mice fed the SOLF diet, whereas, in comparison, iWAT exhibited higher quantities of AA-derived metabolites. Although typically produced in cells through the activity of LOX [7], a noncanonical pathway of mitochondrial oxylipin formation by the activity of CL remodeling enzyme iPLA2y has been described [32]. Furthermore, mitochondrial cytochrome C has been demonstrated to exhibit LOX activity in the presence of calcium, which in turn increases 13-KODE production [33]. Indeed, we saw higher quantities of LOX products, including 13-KODE in BAT of SOLF mice. However, a limitation of this study is that we could not distinguish mitochondria-derived lipid mediators from those produced in other cellular compartments, raising the question of whether these products are derived due to an increase in canonical LOX activity or from noncanonical, mitochondria-dependent pathways.

Due to its mitochondrial density, BAT is highly vascularized to support oxidative metabolism; our finding that plasma fold difference in oxylipins is significantly correlated to those in BAT suggests the likelihood of crosstalk by means of exchange of lipid mediators between these two pools. Recent studies demonstrate that LA-derived 12,13-DiHOME is released from BAT into circulation, and acts to improve insulin sensitivity and metabolic function in skeletal [12] and cardiac muscles [13]. In the current study, 12,13-DiHOME was roughly fourfold higher in both plasma and BAT through the effects of dietary oils alone, even in a low-fat diet, providing a mechanistic link between dietary LA and its reported metabolic health benefits, which is worth further exploration in future studies.

In contrast, iWAT fold difference was not correlated to that of plasma. This is striking due to the functional role of white adipose tissues as an energy storage reservoir as well as the primary regulator of nonesterified fatty acids in circulation. In the current study, our finding that both groups of mice gained weight over the course of the 5-week diet period suggests that there is little exchange between plasma and iWAT lipid mediators when lipids are being stored in the form of triglycerides in the tissue. However, dietary fat quality may have greater influence over oxylipin exchange between these pools in a caloric deficit.

Of the lipid mediators higher in SOLF-diet mice, a striking increase in 13-KODE was observed among all three measured tissues. In human subcutaneous white adipose tissues, 9- and 13-KODE were found to be negatively correlated to HOMA-IR, suggesting that the absence of these oxylipins may predict adipose tissue expansion, inflammation, and insulin resistance [34]. However, very little is known about the functions of 9-and 13-KODE; thus, the relationship between KODEs and metabolic health may be an area of interest for future studies.

In some white adipose tissue depots, the necessity of cyclooxygenase (COX)-1 and COX-2 enzymes, and their resultant prostaglandin products, to activate ‘beige’ thermogenic adipocytes has been demonstrated [35,36]. However, ‘beige’ adipocyte recruitment in iWAT and BAT expansion has been demonstrated by Paschos et al. to be independent of COX-2-mediated processes [37]. In the current study, we observed higher levels of COX-2-derived PGE2 in BAT from SOLF mice. Additionally, the canonical COX-2 metabolite PGF2a was higher in the SOLF diet compared to the POLF diet in both iWAT and BAT. Interestingly, in this same study, the authors identified that COX-2 protein is low in BAT and undetectable in iWAT, suggesting the possibility that PGF2a is produced through an alternative enzymatic pathway in iWAT [37]. Worth noting is that PGF2a also has potent anti-adipogenic effects in white adipose tissues through its regulation of intracellular calcium levels [35]; this relationship is corroborated in the current study, where we identified that PGF2a in iWAT was negatively associated with percent adiposity.

In the current study, we observed higher levels of LOX-mediated HETEs and HODEs derived from AA and LA in iWAT and BAT in the SOLF group. The anti-adipogenic effects of these metabolites were demonstrated in an in vitro study where preadipocytes were treated over the course of their differentiation [38]. These included AA-derived 5-HETE in iWAT, 12-HETE in BAT, and 15-HETE and LA-derived 9- and 13-HODE in both tissues. This suggests the possibility that diet may have an effect to decrease adipose tissue expansion through the activity of LA- and AA-derived LOX metabolites in adipose tissue progenitor cells, which must be addressed in future studies.

The aforementioned 9- and 13-HODE are known agonists of PPAR (α and γ), regulators of adipose tissue differentiation and metabolism. We also saw increases in the known PPARα agonist 8-HETE in iWAT. PPARα expression has been demonstrated to increase adiponectin receptor expression and reduce obesity-related inflammation in adipose tissue [39]. In addition to 9-HODE’s affinity for PPARα, both 9- and 13-HODE have affinity for PPARγ, which not only plays a crucial role in adipogenesis, but is also involved in the expression of the insulin signaling cascade and associated with insulin sensitivity in mature adipocytes [40]. Furthermore, the presence and activation of PPARγ in resident macrophages is integral their proper inflammatory mediation [41], and is required in regulatory T cells for full restoration of adipose tissue insulin sensitivity during thiazolidine diol treatment [42]. Taken together, these studies emphasize that lipid mediators derived from dietary fats may not only modulate endocrine functions of adipose tissue, but may also exert bioactivity on multiple cell types within the adipose tissue milieu, including resident immune cells, providing a functional link to modulation of inflammation.

The findings of the current study underscore that even at low levels in the diet, the types of oils and fatty acids present exhibit the ability to modulate oxylipin and endocannabinoid-like lipid mediators in WAT, BAT and plasma. Furthermore, in healthy animals, diets consisting of linoleic-rich safflower oil resulted in similarities between BAT and plasma. Our review of the known functions of lipid mediators found to be higher in mice consuming the SOLF diet identify several gaps in knowledge where future studies are warranted to investigate the possible links between the functional properties of lipid mediators and potential benefits of LA-rich dietary oils for energy metabolism. Furthermore, this study emphasizes that the utility of lipid mediators expands beyond their autocrine function within adipocytes and underscores the likelihood that paracrine and endocrine functions of many of these metabolites will come to light in future studies.

## Figures and Tables

**Table 1:** (**Part 1 of 2**). **Lipid mediators altered by diet in BAT, iWAT, and plasma of mice consuming POLF and SOLF diets.** (**Part 2 of 2**) **Lipid mediators altered by diet in BAT, iWAT, and plasma of mice consuming POLF and SOLF diets.** Targeted lipidomics was used to identify lipid mediators altered by diet in BAT, iWAT, and plasma. Tabulated are all oxylipins exhibiting significant differences between diet groups in at least one measured tissue, grouped by class and parent fatty acid. Student’s *t*-tests were used to compare the mean oxylipin concentration between diet groups on log-transformed data, with bold values in grey boxes indicating significant differences (*p* < 0.05). Average fold differences were calculated by dividing the average metabolite value for the SOLF diet group by that of the POLF diet group. N = 7–9 mice per group. ND = not detected or low abundance (>25% missing values). Enzymatic pathway abbreviations: COX—cyclooxygenase; LOX—lipoxygenase; Auto—auto-oxidation; CYP—cytochrome P450; sEH—soluble epoxide hydrolase; ADH—alcohol dehydrogenase; PLD—phospholipase D. Full table containing all lipid mediators measured are found in Supplementary Table S2.

		Metabolite	Enzyme	BAT			WAT			Plasma			Average Fold Difference in SOLF vs. POLF		
				POLF Mean ± 95% CI (pmol/g)	SOLF Mean ± 95% CI (pmol/g)	*t*-Test *p*-Value	POLF Mean ± 95% CI (pmol/g)	SOLF Mean ± 95% CI (pmol/g)	*t*-Test *p*-Value	POLF Mean ± 95% CI (nM)	SOLF Mean ± 95% CI (nM)	*t*-Test *p*-Value	BAT	WAT	Plasma
PGs	20:4n6	6-keto-PGF1a	COX1	15.4 ± 4.30	27.5 ± 8.45	**0.01**	13.7 ± 6.94	19.9 ± 6.91	**0.01**	2.84 ± 2.01	2.32 ± 1.79	0.64	**1.76**	**1.46**	0.81
		PGE2	COX2	21.7 ± 7.81	39.2 ± 15.3	**0.03**	21.8 ± 11.6	38.5 ± 14.7	0.15	0.85 ± 0.85	1.12 ± 0.66	0.18	**1.81**	1.81	1.31
		PGF2a	COX2	6.42 ± 1.93	15.2 ± 5.41	**0.006**	5.36 ± 2.17	10.7 ± 2.80	**0.02**	0.97 ± 0.51	2.34 ± 2.06	0.82	**2.37**	**1.99**	1.38
Hydroxyls	18:2n6	13-HODE	LOX	415 ± 164	1801 ± 811	**<0.001**	996 ± 401	3065 ± 755	**<0.001**	16.3 ± 5.60	72.0 ± 36.0	**<0.001**	**4.34**	**3.08**	**4.43**
		9-HODE	LOX	248 ± 76.9	1160 ± 525	**<0.001**	587 ± 232	1762 ± 414	**<0.001**	8.14 ± 3.45	28.9 ± 12.8	**<0.001**	**4.67**	**2.99**	**3.55**
	20:4n6	15-HETE	LOX	32.9 ± 9.63	71.4 ± 17.4	**<0.001**	49.1 ± 13.4	109.4 ± 41.3	**0.009**	8.92 ± 4.75	22.8 ± 20.2	0.21	**2.17**	**2.23**	2.55
		12-HETE	LOX	94.1 ± 37.4	235 ± 90.5	**0.002**	136 ± 65.8	276 ± 132	0.059	584 ± 276	1834 ± 1505	0.16	**2.5**	2.03	3.13
		11-HETE	LOX	14.4 ± 3.65	34.7 ± 10.7	**0.002**	16.3 ± 4.84	31.8 ± 4.57	**<0.001**	7.35 ± 4.07	17.9 ± 15.4	0.25	**2.41**	**1.95**	2.43
		9-HETE	Auto	7.28 ± 1.86	15.3 ± 6.75	0.084	6.81 ± 2.58	13.7 ± 3.26	**0.01**	1.43 ± 0.95	0.79 ± 0.19	0.74	2.1	**2.01**	0.55
		8-HETE	LOX	15.6 ± 8.50	24.3 ± 7.35	0.098	15.8 ± 4.87	36.4 ± 12.09	**0.004**	ND	ND	-	2.09	**2.31**	-
		5-HETE	LOX	19.2 ± 5.03	35.6 ± 12.2	0.053	14.8 ± 3.82	32.9 ± 9.46	**0.005**	16.8 ± 13.7	13.1 ± 1.7	0.80	2.02	**2.23**	0.77
Diols	20:4n6	6-trans-LTB4	LOX	0.55 ± 0.22	0.99 ± 0.41	0.11	0.72 ± 0.30	1.38 ± 0.27	**0.006**	ND	ND	-	1.78	**1.91**	-
	20:5n3	8,15-DiHETE	LOX	7.03 ± 1.60	14.0 ± 5.17	**0.03**	6.81 ± 2.45	15.2 ± 4.09	**0.005**	ND	ND	-	**1.95**	**2.23**	-
Triol	18:2n6	9,12,13-TriHOME (%)	Auto	96.7 ± 33.9	283 ± 148	**0.009**	227 ± 150	294 ± 107	0.28	ND	ND	-	**2.92**	1.29	-
Epoxides	18:2n6	12(13)-EpOME	CYP	16.4 ± 4.71	56.8 ± 19.9	**<0.001**	39.4 ± 17.1	97.4 ± 34.1	**0.01**	1.23 ± 0.33	5.31 ± 1.73	**<0.001**	**3.47**	**2.47**	**4.3**
		9(10)-EpOME	CYP	18.7 ± 5.96	70.6 ± 28.2	**<0.001**	42.4 ± 17.6	117 ± 46.1	**0.007**	0.57 ± 0.26	1.03 ± 0.45	0.25	**3.77**	**2.76**	1.81
		9(10)-EpODE	CYP	1.44 ± 0.44	5.03 ± 3.30	**0.02**	3.72 ± 1.64	20.0 ± 19.2	0.054	ND	ND	-	**3.51**	5.38	-
	20:4n6	14(15)-EpETrE	CYP	3.63 ± 0.77	6.29 ± 1.73	**0.02**	3.85 ± 0.86	5.31 ± 0.81	**0.04**	0.72 ± 0.25	1.17 ± 0.49	0.22	**1.73**	**1.37**	1.62
	11(12)-EpETrE	CYP	2.07 ± 0.52	3.35 ± 1.00	**0.04**	1.88 ± 0.60	2.83 ± 0.49	**0.04**	0.37 ± 0.09	0.56 ± 0.15	0.084	**1.62**	**1.5**	1.49	
Vicinal Diols	18:2n6	12,13-DiHOME	sEH	21.8 ± 4.36	83.0 ± 31.1	**<0.001**	29.8 ± 9.88	108.9 ± 24.3	**<0.001**	8.71 ± 1.84	34.1 ± 12.9	**<0.001**	**3.81**	**3.65**	**3.92**
		9,10-DiHOME	sEH	11.1 ± 1.22	51.2 ± 14.2	**<0.001**	16.4 ± 5.59	66.4 ± 8.56	**<0.001**	3.68 ± 0.63	16.8 ± 4.56	**<0.001**	**4.6**	**4.05**	**4.58**
	18:3n3	15,16-DiHODE	sEH	5.47 ± 2.03	7.10 ± 2.41	0.31	5.60 ± 2.31	10.79 ± 3.20	**0.02**	0.38 ± 0.13	0.51 ± 0.24	0.8	1.3	**1.93**	1.34
		9,10-DiHODE	sEH	0.48 ± 0.07	0.73 ± 0.19	**0.02**	0.61 ± 0.16	1.09 ± 0.18	**0.003**	0.05 ± 0.02	0.07 ± 0.06	0.66	**1.53**	**1.8**	1.65
		14,15-DiHETrE	sEH	1.58 ± 0.54	2.68 ± 0.54	**0.004**	0.93 ± 0.15	2.23 ± 0.32	**<0.001**	1.57 ± 0.14	3.26 ± 0.70	**<0.001**	**1.7**	**2.4**	**2.08**
	20:4n6	11,12-DiHETrE	sEH	1.14 ± 0.36	1.64 ± 0.38	0.07	0.55 ± 0.11	1.28 ± 0.23	**<0.001**	0.91 ± 0.12	1.80 ± 0.38	**<0.001**	1.44	**2.34**	**1.99**
		8,9-DiHETrE	sEH	1.83 ± 0.94	2.41 ± 0.58	0.11	1.02 ± 0.24	1.57 ± 0.40	**<0.001**	2.68 ± 0.40	3.54 ± 0.60	**0.03**	1.31	1.54	**1.32**
		5,6-DiHETrE	sEH	0.41 ± 0.16	0.48 ± 0.16	0.57	0.19 ± 0.06	0.35 ± 0.10	**0.02**	1.51 ± 0.26	2.15 ± 0.45	**0.04**	1.16	**1.83**	**1.42**
	22:6n3	19,20-DiHDoPA	sEH	4.68 ± 2.34	4.08 ± 0.93	0.98	1.86 ± 0.48	2.67 ± 0.48	**0.04**	2.47 ± 0.36	2.08 ± 0.44	0.18	0.87	**1.44**	0.84
				**BAT**			**WAT**			**Plasma**			**Average fold difference in SOLF vs. POLF**		
		**Metabolite**	**Enzyme**	**POLF Mean ± 95% CI (pmol/g)**	**SOLF Mean ± 95% CI (pmol/g)**	***t*-Test *p*-Value**	**POLF Mean ± 95% CI (pmol/g)**	**SOLF Mean ± 95% CI (pmol/g)**	***t*-Test *p*-Value**	**POLF Mean ± 95% CI (nM)**	**SOLF Mean ± 95% CI (nM)**	***t*-Test *p*-Value**	**BAT**	**WAT**	**Plasma**
Ketones	18:2n6	13-KODE	ADH	68.2 ± 37.5	390 ± 288	**0.01**	262 ± 169	1898 ± 1961	**0.03**	1.14 ± 0.47	3.71 ± 1.74	**0.02**	**5.73**	**7.25**	**3.25**
		9-KODE	ADH	113 ± 71.1	727 ± 565	**0.04**	472 ± 322	2227 ± 1547	**0.046**	1.24 ± 0.44	6.97 ± 2.45	**0.007**	**6.44**	**4.72**	**5.61**
		12(13)-Ep-9-KODE	ADH	60.4 ± 16.3	163 ± 102	**0.04**	180 ± 110	493 ± 237	0.065	ND	ND	-	**2.7**	2.74	-
	20:4n6	15-KETE	ADH	4.24 ± 1.73	13.1 ± 4.91	**0.008**	5.96 ± 2.82	12.7 ± 4.11	**0.04**	ND	ND	-	**3.08**	**2.13**	-
		5-KETE	ADH	6.97 ± 2.57	11.6 ± 4.79	0.25	5.57 ± 4.15	9.80 ± 2.18	**0.02**	ND	ND	-	1.67	**1.78**	
N-Acylethanolamines	16:00	PEA	PLD	331 ± 318	448 ± 219	0.29	189 ± 144	746 ± 375	**0.007**	10.5 ± 1.69	9.56 ± 2.10	0.44	1.35	**3.94**	0.91
	18:1n9	OEA	PLD	559 ± 236	371 ± 109	0.2	287 ± 42.6	521 ± 206	0.08	22.9 ± 4.51	14.1 ± 3.78	**0.03**	0.66	1.82	**0.62**
	18:2n6	LEA	PLD	143 ± 61.3	322 ± 127	**0.02**	42.5 ± 4.86	127 ± 13.7	**0**	4.40 ± 0.66	9.87 ± 1.95	**<0.001**	**2.25**	**2.99**	**2.25**
	20:3n6	DGLEA	PLD	2.66 ± 0.85	3.02 ± 0.54	0.31	1.77 ± 0.37	3.48 ± 0.93	**0.005**	0.14 ± 0.03	0.17 ± 0.06	0.76	1.13	**1.97**	1.15
	20:4n6	AEA	PLD	34.7 ± 18.3	36.1 ± 15.8	0.6	8.40 ± 1.23	11.1 ± 1.25	**0.007**	1.68 ±0.29	1.77 ± 0.44	0.89	1.04	**1.32**	1.05
	22:5n6	DEA	PLD	1.38 ± 0.39	2.21 ± 0.51	**0.02**	1.11 ± 0.40	1.99 ± 0.31	**0.006**	0.21 ± 0.10	0.42 ± 0.14	**<0.001**	**1.6**	**1.78**	**2.03**
NEFA (rel abs)	18:2n6	LA	-	4.88 ± 1.04	8.01 ± 1.70	**0.005**	5.69 ± 1.42	10.1 ± 2.74	**<0.001**	0.032 + 0.007	0.070 + 0.014	**<0.001**	**1.64**	**1.78**	**2.18**
	20:4n6	AA	-	9.35 ± 2.14	12.4 ± 3.11	0.13	5.52 ± 1.19	9.57 ± 1.10	**<0.001**	0.033 ± 0.007	0.065 ± 0.010	**<0.001**	1.33	**1.73**	**1.99**
MAG	18:1n9	1/2-OG	-	13,600 ± 6340	7120 ± 2260	0.18	5610 ± 2400	3900 ± 603	0.74	9905 ± 1670	4290 ± 608	**<0.001**	0.56	0.7	**0.43**
	18:2n6	1/2-LG	-	4120 ± 1790	13,200 ± 2970	**<0.001**	2640 ± 1,420	5310 ± 1760	**0.02**	1640 ± 305	5120 ± 1050	**<0.001**	**3.19**	**2.01**	**3.12**
	20:4n6	1/2-AG	-	1250 ± 307	1400 ± 408	0.57	7.08 ± 0.41	2540 ± 1993	0.38	118 ± 23.7	158 ± 24.2	**0.046**	1.12	1.93	**1.34**
Gly	18:1n9	NO-Gly	-	5.84 ± 2.42	4.71 ± 1.59	0.75	5.52 ± 2.53	5.99 ± 1.06	0.39	2.29 ± 0.48	1.45 ± 0.50	**0.03**	1.08	0.8	**0.63**
	20:4n6	NA-Gly	-	ND	ND	-	ND	ND	-	0.23 ± 0.05	0.39 ± 0.11	**0.02**	-	-	**1.73**

## Data Availability

The data presented in this study are available in article and Supplementary Material.

## Supplementary Materials

The following supporting information can be downloaded at: https://www.mdpi.com/article/10.3390/metabo12080743/s1. Table S1: Comparison of AIN-93G diets containing palm oil (POLF) or linoleic-rich safflower oil (SOLF) as the fat source; Table S2 (parts 1 and 2): All lipid mediators measured in BAT, iWAT, and plasma of mice consuming POLF and SOLF diets; Table S3: Spearman’s rank order correlation coefficients for diet-independent changes to lipid mediators and physiological measurements; Table S4: Percent of total fold difference by enzymatic pathway in BAT, WAT, and plasma; Table S5: average fold difference within each enzymatic pathway in BAT, WAT, and plasma.
