# Supplementary material for: Linoleate-Rich Safflower Oil Diet Increases Linoleate-Derived Bioactive Lipid Mediators in Plasma, and Brown and White Adipose Depots of Healthy Mice
Source: Metabolites. 2022 Aug 12;12(8):743. doi: 10.3390/metabo12080743 (PMC9412644; doi:10.3390/metabo12080743)
Supplement: Supplementary file 1 [file metabolites-12-00743-s001.zip › 2022-07-15 Supplementary Tables S1 S2 S4 S5.pdf]

Table S1. Comparison of AIN-93G diets containing palm oil (POLF) or linoleate-rich safflower oil (SOLF) as the fat source.

	POLF Diet		SOLF Diet	
Macronutrient	Gram %	Kcal %	Gram %	Kcal %
Protein	15	15	15	15
Carbohydrate	71	71	71	71
Fat	6	14	6	14
Total		100	3.95	
Kcal/gram	3.95			100
Ingredient (per kilogram)	Gram	Kcal	Gram	Kcal
Casein	145	580	145	580
L-Cysteine	1.8	1762	1.8	7
Corn starch	440.588	620	440.588	1762
Maltodextrin	155	400	155	620
Sucrose	100	0	100	400
Cellulose	50	540	50	0
Palm oil	60	0	0	0
Safflower oil, high-linoleic	0	0	60	540
t-butylhydroquinone	.012	0	.012	0
Mineral mix	35	0	35	0
Vitamin mix	10	40	10	40
Choline bitartrate	2.5	0	2.5	0
Yellow dye #5	0.1	0	0.05	0
Red dye #40	0	0	0.05	0
Blue dye #1	1	0	0	0
Total	1000	3950	1000	3950

		Metabolite	Enzyme	BAT POLF Mean ± 95% CI (pmol/g)	BAT SOLF Mean ± 95% CI (pmol/g)	BAT T-test P-value	WAT POLF Mean ± 95% CI (pmol/g)	WAT SOLF Mean ± 95% CI (pmol/g)	WAT T-test P-value	Plasma POLF Mean ± 95% CI (nM)	Plasma SOLF Mean ± 95% CI (nM)	Plasma T-test P-value	Average fold difference in SOLF vs POLF: BAT	WAT	Plasma
PGs	20:3n6	PGE1	COX2	1.68 ± 0.85	3.33 ± 2.10	0.17	1.74 ± 0.94	3.28 ± 1.81	0.21	ND	ND	-			
	20:4n6	6-keto-PGF1a	COX1	15.4 ± 4.30	27.5 ± 8.45	**0.01**	13.7 ± 6.94	19.9 ± 6.91	**0.01**	2.84 ± 2.01	2.32 ± 1.79	0.64	**1.79**	**1.45**	0.81
		PGE1	COX2	1.77 ± 0.79	3.34 ± 2.10	0.065	1.74 ± 0.94	3.28 ± 1.81	0.34	ND	ND	-	1.88	1.89	-
		PGE2	COX2	21.7 ± 7.81	39.2 ± 15.3	**0.03**	21.27 ± 11.6	38.5 ± 14.7	0.15	0.85 ± 0.85	1.12 ± 0.66	0.18	**1.81**	1.81	1.31
		PGD2	COX2	10.7 ± 4.14	16.5 ± 10.2	0.31	11.2 ± 5.15	23.7 ± 15.0	0.25	ND	ND	-	1.54	2.13	-
		15-deoxy-PGJ2	COX2	2.01 ± 0.34	2.11 ± 0.48	0.87	2.33 ± 0.51	2.23 ± 0.46	0.7	ND	ND	-	1.05	0.96	-
		F2 isoprostanes	COX2	2.27 ± 0.52	2.59 ± 0.61	0.56	2.13 ± 0.46	2.22 ± 0.78	0.75	5.31 ±1.95	7.36 ± 1.67	0.15	1.78	1.04	1.38
		PGF2a	COX2	6.42 ± 1.93	15.2 ± 5.41	**0.006**	5.36 ± 2.17	10.7 ± 2.80	**0.02**	0.97 ± 0.51	2.34 ± 2.06	0.82	**2.37**	**1.99**	1.38
Hydroxyls	18:2n6	13-HODE	LOX	415 ± 164	1801 ± 811	**<.001**	996 ± 401	3065 ± 755	**<.001**	16.3 ± 5.60	72.0 ± 36.0	**<.001**	**4.34**	**3.08**	**4.43**
		9-HODE	LOX	248 ± 76.9	1160 ± 525	**<.001**	587 ± 232	1762 ± 414	**<.001**	8.14 ± 3.45	28.9 ± 12.8	**<.001**	**4.68**	**3.00**	**3.55**
	18:3n3	13-HOTE	LOX	23.5 ± 13.6	21.9 ± 8.70	0.64	84.3 ± 55.2	92.2 ± 56.6	0.94	0.53 ± 0.17	0.81 ± 0.16	0.061	0.93	1.09	1.53
		9-HOTE	LOX	2.59 ± 0.76	4.17 ± 2.29	0.34	6.32 ± 2.81	7.72 ± 2.66	0.44	0.15 ± 0.06	0.17 ± .06	0.62	1.61	1.22	1.14
	20:4n6	15-HETE	LOX	32.9 ± 9.63	71.4 ± 17.4	**<.001**	49.1 ± 13.4	109 ± 41.3	**0.009**	8.92 ± 4.75	22.8 ± 20.2	0.21	**2.17**	**2.22**	2.55
		5-HETE	LOX	94.1 ± 37.4	235 ± 90.5	**0.002**	136 ± 65.8	276 ± 132	0.059	584 ± 276	1834 ± 1505	0.16	**2.50**	2.03	3.13
		11-HETE	LOX	14.4 ± 3.65	34.7 ± 10.7	**0.002**	16.3 ± 4.84	31.8 ± 4.57	**<.001**	7.35 ± 4.07	17.9 ± 15.4	0.25	**2.41**	**1.95**	2.43
		9-HETE	Auto	7.28 ± 1.86	15.3 ± 6.75	0.084	6.81 ± 2.58	13.7 ± 3.26	**0.01**	1.43 ± 0.95	0.79 ± 0.19	0.74	**2.1**	**2.00**	0.55
		8-HETE	LOX	15.6 ± 8.50	24.3 ± 7.35	0.098	15.8 ± 4.87	36.4 ± 12.09	**0.004**	ND	ND	-	2.09	**2.30**	-
		5-HETE	LOX	19.2 ± 5.03	35.6 ± 12.2	0.053	14.8 ± 3.82	32.9 ± 9.46	**0.005**	16.8 ± 13.7	13.1 ± 1.7	0.8	**2.00**	**2.20**	0.77
		15-HETE-EA	LOX	.053 ± .056	.069 ± .040	0.35	.030 ± .019	.116 ± .054	0.052	ND	ND	-	1.31	3.95	-
	20:5n3	15-HEPE	LOX	1.40 ± 0.70	1.62 ± 0.61	0.34	4.71 ± 2.60	5.82 ± 4.52	0.95	ND	ND	-	1.86	2.23	-
		12-HEPE	LOX	1.27 ± 0.47	1.78 ± 0.63	0.14	2.30 ± 0.99	2.69 ± 1.33	0.9	4.80 ± 2.15	7.93 ± 6.62	0.14	1.65	1.4	1.17
		9-HEPE	LOX	0.54 ± 0.18	0.47 ± 0.24	0.56	0.44 ± 0.25	0.82 ± 0.45	0.26	ND	ND	-	1.4	1.17	-
		5-HEPE	LOX	1.07 ± 0.17	0.86 ± 0.33	0.18	0.53 ± 0.23	1.01 ± 0.50	0.24	0.99 ± 0.83	0.60 ±0.34	0.55	0.61	0.8	1.9
	22:6n3	17-HDoHE	LOX	26.5± 12.0	32.7 ± 14.5	0.46	75.3 ± 35.2	106 ± 69.9	0.69	3.95 ± 2.48	3.83 ± 1.48	0.87	1.23	1.41	0.97
		14-HDoHE	LOX	42.9 ± 18.3	49.0 ± 23.1	0.64	106 ± 53.7	143 ± 96.5	0.7	68.7 ± 27.0	146 ± 86.7	0.18	1.14	1.35	2.13
		4-HDoHE	LOX	2.32 ± 0.50	2.73 ± 1.09	0.96	3.28 ± 1.16	2.92 ± 0.84	0.65	2.84 ± 1.45	2.03 ± 0.52	0.36	1.18	0.89	0.72
Diols	20:4n6	LTB4	LOX	ND	ND	-	ND	ND	-	0.44 ± 0.39	0.28 ± 0.19	0.6	-	-	0.64
		6-trans-LTB4	LOX	0.55 ± 0.22	0.99 ± 0.41	0.11	0.72 ± 0.30	1.38 ± 0.27	**0.006**	ND	ND	-	1.78	**1.9**	-
	20:5n3	8,15-DiHETE	LOX	7.03 ± 1.60	13.97 ± 5.17	**0.03**	6.81 ± 2.45	15.2 ± 4.09	**0.005**	ND	ND	-	**2**	**2.2**	-
	22:6n3	Protectin DX	LOX	0.31 ± 0.11	0.37 ± 0.07	0.21	0.46 ± 0.15	0.76 ± 0.33	0.26	ND	ND	-	1.18	1.66	-
Triol	18:2n6	9,12,13-TriHOME (%)	Auto	96.7 ± 33.9	283 ± 148	**0.009**	227 ± 150	294 ± 107	0.28	ND	ND	-	**2.9**	1.29	-
Epoxides	18:2n6	12(13)-EpOME	CYP	16.4 ± 4.71	56.8 ± 19.9	**0**	39.4 ± 17.1	97.4 ± 34.1	**0.01**	1.23 ± 0.33	5.31 ± 1.73	**<.001**	**3.5**	**2.5**	**4.3**
		9(10)-EpOME	CYP	18.7 ± 5.96	70.6 ± 28.2	**<.001**	42.4 ± 17.6	117 ± 46.1	**0.007**	0.57 ± 0.26	1.03 ± 0.45	0.25	**3.8**	**2.8**	1.81
	18:3n3	15(16)-EpODE	CYP	2.62 ± 0.68	3.03 ± 0.62	0.34	3.64 ± 1.35	7.11 ± 4.73	0.21	2.12 ± 0.49	2.87 ± 0.99	0.2	1.16	1.95	1.35
		12(13)-EpODE	CYP	0.32 ± 0.10	0.27 ± 0.25	0.11	0.74 ± 0.28	0.84 ± 0.36	0.71	ND	ND	-	0.84	1.13	-
		9(10)-EpODE	CYP	1.44 ± 0.44	5.03 ± 3.30	**0.02**	3.72 ± 1.64	20.0 ± 19.2	0.054	ND	ND	-	**3.5**	5.38	-
	20:4n6	14(15)-EpETrE	CYP	3.63 ± 0.77	6.29 ± 1.73	**0.02**	3.85 ± 0.86	5.31 ± 0.81	**0.04**	0.72 ± 0.25	1.17 ± 0.49	0.22	**1.7**	**1.4**	1.62
		11(12)-EpETrE	CYP	2.07 ± 0.52	3.35 ± 1.00	**0.04**	1.88 ± 0.60	2.83 ± 0.49	**0.04**	0.37 ± 0.09	0.56 ± 0.15	0.084	**1.6**	**1.5**	1.49
		8(9)-EpETrE	CYP	5.90 ± 1.70	5.49 ± 1.83	0.59	8.34 ± 4.05	7.48 ± 3.29	0.66	ND	ND	-	0.93	0.73	-
	22:6n3	19(20)-EpDPE	CYP	3.17 ± 1.01	2.21 ± 0.60	0.15	3.55 ± 1.02	2.62 ± 0.53	0.14	3.98 ± 0.75	3.82 ± 1.17	0.63	0.7	0.74	0.96
		16(17)-EpDPE	CYP	0.87 ± 0.32	0.91 ± 0.40	0.65	1.36 ± 0.68	0.68 ± 0.26	0.28	ND	ND	-	1.04	0.51	-

Supplementary Table S2 (1 of 2)

Supplementary Table S2 (2 of 2). All lipid mediators measured in BAT, iWAT, and plasma of mice consuming POLF and SOLF diets. Targeted lipidomics was used to identify lipid mediators altered by diet in BAT, iWAT, and plasma. Lipid mediators are grouped by class and parent fatty acid. Average fold difference was calculated by dividing the average metabolite value for the SOLF diet group by that of the POLF diet group. A student's t-test was used to compare the mean oxylipin concentration between diet groups on log-transformed data, with bolded values in grey boxes indicating significant differences (P<.05). N=7-9 mice per group. ND= not detected or low abundance (>25% missing values). Enzymatic pathway abbreviations: COX – cyclooxygenase; LOX – lipoxygenase; Auto- auto-oxidation; CYP – cytochrome P450; sEH – soluble epoxide hydrolase; ADH – alcohol dehydrogenase; PLD – phospholipase D.

				BAT			WAT			Plasma			Average fold difference in SOLF vs POLF		
		Metabolite	Enzyme	POLF Mean ± 95% CI (pmol/g)	SOLF Mean ± 95% CI (pmol/g)	T-test P-value	POLF Mean ± 95% CI (pmol/g)	SOLF Mean ± 95% CI (pmol/g)	T-test P-value	POLF Mean ± 95% CI (nM)	SOLF Mean ± 95% CI (nM)	T-test P-value	BAT	WAT	Plasma
Vicinal Diols	**18:2n6**	12,13-DiHOME	sEH	21.8 ± 4.36	83.0 ± 31.1	**0**	29.8 ± 9.88	108.9 ± 24.3	**0.0001**	8.71 ± 1.84	34.1 ± 12.9	**0**	**3.8**	**3.7**	**3.92**
		9,10-DiHOME	sEH	11.1 ± 1.22	51.2 ± 14.2	**0**	16.4 ± 5.59	66.4 ± 8.56	**0**	3.68 ± 0.63	16.8 ± 4.56	**0**	**4.6**	**4.1**	**4.58**
	**18:3n3**	15,16-DiHODE	sEH	5.47 ± 2.03	7.10 ± 2.41	0.31	5.60 ± 2.31	10.79 ± 3.20	**0.02**	0.38 ± 0.13	0.51 ± 0.24	0.8	1.3	**1.9**	1.34
		9,10-DiHODE	sEH	0.48 ± 0.07	0.73 ± 0.19	**0.02**	0.61 ± 0.16	1.09 ± 0.18	**0.003**	0.05 ± 0.02	0.07 ± 0.06	0.66	**1.5**	**1.8**	1.65
		14,15-DiHETrE	sEH	1.58 ± 0.54	2.68 ± 0.54	**0.004**	0.93 ± 0.15	2.23 ± 0.32	**0**	1.57 ± 0.14	3.26 ± 0.70	**0**	**1.7**	**2.4**	**2.08**
	**20:4n6**	11,12-DiHETrE	sEH	1.14 ± 0.36	1.64 ± 0.38	0.07	0.55 ± 0.11	1.28 ± 0.23	**0.0002**	0.91 ± 0.12	1.80 ± 0.38	**0**	1.44	**2.3**	**1.99**
		8,9-DiHETrE	sEH	1.83 ± 0.94	2.41 ± 0.58	0.11	1.02 ± 0.24	1.57 ± 0.40	0.13	2.68 ± 0.40	3.54 ± 0.60	**0.03**	1.31	1.54	**1.32**
		5,6-DiHETrE	sEH	0.41 ± 0.16	0.48 ± 0.16	0.57	0.19 ± 0.06	0.35 ± 0.10	**0.02**	1.51 ± 0.26	2.15 ± 0.45	**0.04**	1.16	**1.8**	**1.42**
	**22:6n3**	19,20-DiHDoPA	sEH	4.68 ± 2.34	4.08 ± 0.93	0.98	1.86 ± 0.48	2.67 ± 0.48	**0.04**	2.47 ± 0.36	2.08 ± 0.44	0.18	0.87	**1.4**	0.84
Ketones	**18:2n6**	13-KODE	ADH	68.2 ± 37.5	390 ± 288	**0.01**	262 ± 169	1898 ± 1961	**0.03**	1.14 ± 0.47	3.71 ± 1.74	**0.02**	**5.7**	**7.3**	**3.25**
		9-KODE	ADH	113 ± 71.1	727 ± 565	**0.04**	472 ± 322	2227 ± 1547	**0.046**	1.24 ± 0.44	6.97 ± 2.45	**0.01**	**6.4**	**4.7**	**5.61**
		12(13)-Ep-9-KODE	ADH	60.4 ± 16.3	163 ± 102	**0.04**	180 ± 110	493 ± 237	0.065	ND	ND	-	**2.7**	2.74	-
	**20:4n6**	15-KETE	ADH	4.24 ± 1.73	13.06 ± 4.91	**0.008**	5.96 ± 2.82	12.7 ± 4.11	**0.04**	ND	ND	-	**3.1**	**2.1**	-
		5-KETE	ADH	6.97 ± 2.57	11.63 ± 4.79	0.25	5.57 ± 4.15	9.80 ± 2.18	**0.02**	ND	ND	-	1.67	**1.8**	
N-Acylethanolamines	**16:00**	PEA	PLD	331 ± 318	448 ± 219	0.29	189 ± 144	746 ± 375	**0.007**	10.54 ± 1.69	9.56 ± 2.10	0.44	1.35	**3.9**	0.91
	**18:00**	SEA	PLD	ND	ND	-	ND	ND	-	3.80 ± 1.08	4.07 ± 1.10	0.69	-	-	1.07
	**16:1n7**	POEA (rel abs)	PLD	127 ± 37	87.2 ± 19.8	0.089	92.1 ± 13.8	112 ± 31.6	0.3486	0.05 ± 0.01	0.03 ± 0.00	0.0029			
	**18:1n9**	OEA	PLD	559 ± 236	371 ± 109	0.2	287 ± 42.6	521 ± 206	0.08	22.85 ± 4.51	14.1 ± 3.78	**0.03**	0.66	1.82	**0.62**
	**18:2n6**	LEA	PLD	143 ± 61.3	322 ± 127	**0.02**	42.5 ± 4.86	127 ± 13.7	**0**	4.40 ± 0.66	9.87 ± 1.95	**0**	**2.3**	**3**	**2.25**
	**18:3n3**	aLEA	PLD	0.51 ± 0.12	0.60 ± 0.19	0.54	0.45 ± 0.23	0.68 ± 0.26	0.14	0.03 ± 0.02	0.05 ± 0.04	0.57	1.17	1.5	1.5
	**20:3n6**	DGLEA	PLD	2.66 ± 0.85	3.02 ± 0.54	0.31	1.77 ± 0.37	3.48 ± 0.93	**0.005**	0.14 ± 0.03	0.17 ± 0.06	0.76	1.13	**2**	1.15
	**20:4n6**	AEA	PLD	34.7 ± 18.3	36.1 ± 15.8	0.6	8.40 ± 1.23	11.13 ± 1.25	**0.007**	1.68 ±0.29	1.77 ± 0.44	0.89	1.04	**1.3**	1.05
	**20:5n3**	EPEA	PLD	54.8 ± 17.1	34.2 ± 13.4	0.086	33.9 ± 15.2	43.3 ± 22.2	0.75	ND	ND	-	0.62	1.27	-
	**22:5n6**	DEA	PLD	1.38 ± 0.39	2.21 ± 0.51	**0.02**	1.11 ± 0.40	1.99 ± 0.31	**0.006**	0.21 ± 0.10	0.42 ± 0.14	**0.01**	**1.6**	**1.8**	**2.03**
	**22:6n3**	DHEA	PLD	12.7 ± 4.50	11.04 ± 2.47	0.81	6.64 ± 1.01	5.97 ± 1.08	0.31	1.46 ± 0.23	1.21 ± 0.24	0.17	0.87	0.9	0.82
NEFA (rel abs)	18:2n6	LA	-	4.88 ± 1.04	8.01 ± 1.70	**0.005**	5.69 ± 1.42	10.1 ± 2.74	**<.001**	.032 + .007	.070 + .014	**<.001**	**1.6**	**1.8**	**2.18**
	18:3n3	ALA	-	12.8 + 3.14	12.5 + 5.12	0.68	25.7 + 11.8	31.9 + 21.5	0.81	.041 ± .009	.055 + .027	0.36	1	1.2	1.34
	20:4n6	AA	-	9.35 ± 2.14	12.4 ± 3.11	0.13	5.52 ± 1.19	9.57 ± 1.10	**<.001**	.033 ± .007	.065 ± .010	**<.001**	1.3	**1.7**	**1.99**
	20:5n3	EPA	-	33.9 ± 7.89	29.4 ± 8.20	0.45	34.9 ± 15.8	30.4 ± 9.60	0.75	.051 ± .011	.052 ± .020	0.91	0.9	0.9	1.03
	22:5n3	DHA	-	8.73 ± 3.26	7.26 ± 1.27	0.59	5.74 ± 1.42	5.80 ± 0.88	0.87	.045 ± .009	.048 ± .010	0.72	0.8	1	1.06
MAG	18:1n9	1/2-OG	-	13600 ± 6340	7120 ± 2260	0.18	5610 ± 2400	3900 ± 603	0.74	9905 ± 1670	4290 ± 608	**<.001**	0.6	0.7	**0.43**
	18:2n6	1/2-LG	-	4120 ± 1790	13200 ± 2970	**<.001**	2640 ± 1,420	5310 ± 1760	**0.02**	1640 ± 305	5120 ± 1050	**<.001**	**3.2**	**2**	**3.12**
	20:4n6	1/2-AG	-	1250 ± 307	1400 ± 408	0.57	7.08 ± 0.41	2540 ± 1993	0.38	118 ± 23.7	158 ± 24.2	**0.046**	1.1	1.9	**1.34**
Gly	18:1n9	NO-gly	-	5.84 ± 2.42	4.71 ± 1.59	0.75	5.52 ± 2.53	5.99 ± 1.06	0.39	2.29 ± 0.48	1.45 ± 0.50	**0.03**	1.1	0.8	**0.63**
	20:4n6	NA-Gly	-	ND	ND	-	ND	ND	-	0.23 ± 0.05	0.39 ± 0.11	**0.02**	-	-	**1.73**

Table S3 can be found in an excel spreadsheet labeled 'Supplementary Table S3'.

Table S4. Percent of total fold difference by enzymatic pathway. The sum of all fold differences (SOLF/POLF) computed in univariate analyses for those metabolites with significant fold differences within one of the three tissues measured was taken. The contribution of each pathway as a percent of the total fold difference was calculated for each tissue.

	BAT	iWAT	Plasma
Sum of fold differences in SOLF v. POLF diets	94.36	96.42	74.48
% Auto	5.3	3.4	0.7
% COX	6.3	5.5	4.7
% CYP	14.9	14.0	23.6
% sEH	18.8	21.8	25.7
% PLD	8.5	14.3	10.8
% LOX	46.2	41.0	34.5
**Total %**	**100.0**	**100.0**	**100.0**

Supplementary Table S5. Average fold difference within each enzymatic pathway. The mean (+/- SEM) was calculated using each average fold difference (SOLF/POLF) computed in univariate analyses for those metabolites with significant fold differences within one of the three tissues measured. A 1-Way ANOVA with Tukey's post hoc test was used to determine differences in average fold difference between tissues, with P-values <.05 being significant. Letters in the subscript indicate significant (P<.05) differences between tissues as determined by Tukey's post-hoc test.

	average fold difference (POLF/SOLF) of metabolites within enzymatic pathway			
Enzymatic pathway	BAT	iWAT	Plasma	P-value
Auto-oxidation	2.51 ± 0.41[a]	1.65 ± 0.36[ab]	0.55 ± 0.275[b]	**.04**
COX	1.98 ± 0.20 [a]	1.75 ± 0.16 [ab]	0.275 ± 0.275 [b]	**.04**
CYP	2.82 ± 0.47	2.70 ± 0.72	3.51 ± 1.31	.57
sEH	1.97 ± 0.43	2.33 ± 0.31	2.13 ± 0.27	.81
PLD	1.34 ± 0.22	2.30 ± 0.40	1.36 ± 0.27	.063
LOX	3.11 ± 0.41	2.83 ± 0.40	3.22 ± 0.38	.12
